# The growth factor progranulin attenuates neuronal injury induced by cerebral ischemia-reperfusion through the suppression of neutrophil recruitment

**DOI:** 10.1186/1742-2094-10-105

**Published:** 2013-08-23

**Authors:** Yusuke Egashira, Yukiya Suzuki, Yukio Azuma, Toshinori Takagi, Keisuke Mishiro, Sou Sugitani, Kazuhiro Tsuruma, Masamitsu Shimazawa, Shinichi Yoshimura, Masanori Kashimata, Toru Iwama, Hideaki Hara

**Affiliations:** 1 Department of Biofunctional Evaluation, Molecular Pharmacology, Gifu Pharmaceutical University, 1-25-4 Daigaku-nishi, Gifu 501-1196, Japan; 2 Department of Neurosurgery, Gifu University Graduate School of Medicine, 1-1 Yanagido, Gifu 501-1194, Japan; 3 Department of Dental Pharmacology, Asahi University School of Dentistry, 1851 Hozumi, Mizuho, Gifu 501-0296, Japan

**Keywords:** Cerebral ischemia-reperfusion, Inflammation, Progranulin, Neuroprotection, Neutrophil recruitment

## Abstract

**Background:**

To improve the clinical outcome of patients who suffered ischemic stroke, cerebral ischemia-reperfusion (I/R) injury is one of the major concerns that should be conquered. Inflammatory reactions are considered a major contributor to brain injury following cerebral ischemia, and I/R exacerbates these reactions. The aim of this study was to investigate the possible ameliorative effects of progranulin (PGRN) against I/R injury in mice.

**Methods:**

*In vivo* I/R was induced in four-week-old male ddY mice by 2 h of MCAO (middle cerebral artery occlusion) followed by 22 h of reperfusion. We evaluate expression of PGRN in I/R brain, efficacy of recombinant-PGRN (r-PGRN) treatment and its therapeutic time-window on I/R injury. Two hours after MCAO, 1.0 ng of r-PRGN or PBS was administered via intracerebroventricular. We assess neutrophil infiltration, expression of tumor necrosis factor (TNF)-α, matrix metalloproteinase-9 (MMP-9) and phosphorylation of nuclear factor-κB (NF-κB) by immunofluorescense staining and Western blotting. We also investigate neutrophil chemotaxis and intercellular adhesion molecule-1 (ICAM-1) expression *in vitro* inflammation models using isolated neutrophils and endothelial cells.

**Results:**

We found that expression of PGRN was decreased in the I/R mouse brain. r-PGRN treatment at 2 h after MCAO resulted in a reduction in the infarct volume and decreased brain swelling; this led to an improvement in neurological scores and to a reduction of mortality rate at 24 h and 7 d after MCAO, respectively. Immunohistochemistry, Western blotting, and gelatin zymography also confirmed that r-PGRN treatment suppressed neutrophil recruitment into the I/R brain, and this led to a reduction of NF-κB and MMP-9 activation. In the *in vitro* inflammation models, PGRN suppressed both the neutrophil chemotaxis and ICAM-1 expression caused by TNF-α in endothelial cells.

**Conclusions:**

PGRN exerted ameliorative effects against I/R-induced inflammation, and these effects may be due to the inhibition of neutrophil recruitment into the I/R brain.

## Introduction

Stroke is a devastating disease and a leading cause of death and severe disability worldwide [1]. Although the majority of strokes are ischemic, few curative therapeutic strategies are available for patients who have suffered an ischemic stroke. At present, restoration of cerebral blood flow is the best and, indeed, the only strategy available to rescue the brain tissue from infarction, and tissue-type plasminogen activator is, thus far, the only clinically approved treatment for acute ischemic stroke. However, if the time of reperfusion exceeds the therapeutic time window, the risk of cerebral ischemia-reperfusion (I/R) injury increases paradoxically. Severe I/R injury can cause fatal hemorrhagic transformation or brain swelling, which leads to poorer clinical outcomes.

Inflammatory reactions are considered a major contributor to brain injury following cerebral ischemia [2]. It has been reported that cerebral ischemia triggers these inflammatory reactions around the ischemic brain, and that subsequent reperfusion exacerbates them [3]. Experimentally and clinically, proinflammatory mediators, such as tumor necrosis factor (TNF)-α, are rapidly released from injured tissue in the acute phase of cerebral ischemia [4,5]; this induces the recruitment and activation of inflammatory cells, including various types of leukocytes [6,7]. This is one of the key features of the neuroimmunological reaction to cerebral ischemia [2,8]. Among the various types of leukocytes, neutrophils are the first to infiltrate into the ischemic brain, and they peak one to three days following focal cerebral ischemia [4,9]. During these early phases of post-cerebral I/R, infiltrating neutrophils cause critical pathological changes via several mechanisms, including the release of elastase, excessive production of reactive oxygen species (ROS), and induction of matrix metalloproteinase-9 (MMP-9) [7,10,11]. Inhibition of neutrophil infiltration, therefore, represents a potential anti-inflammatory strategy for neuroprotection in the acute stages of ischemic stroke [7].

Progranulin (PGRN) is a 593-amino acid, 68.5-kDa cysteine-rich protein that is typically secreted in a highly glycosylated 88-kDa form [12], and is known to perform various biological functions, such as the regulation of cell growth, embryonic development and tissue repair [13,14]. Recently, it was reported that PGRN directly binds to TNF receptors and suppresses TNF-α-mediated inflammation in a mouse model of rheumatoid arthritis [15]. In the central nervous system (CNS), PGRN has been reported to function as a neurotrophic factor [16], and decreased PGRN expression due to null mutations of the *PGRN* gene is thought to be associated with frontotemporal lobar dementia [12,17]. Although these previous studies suggest the interactions between inflammatory pathogenesis in the CNS and the potential role of PGRN, the anti-inflammatory actions and the therapeutic prospects of PGRN in acute neuronal injury have not been investigated well [18].

In the present study, we examined whether PGRN has potential ameliorative effects against brain I/R injury, and also investigated the underlying mechanisms, mainly focusing on the anti-inflammatory actions of PGRN by using an experimental model of focal cerebral ischemia-reperfusion.

## Materials and methods

### Animals

All animal protocols were conducted in accordance with the “Animal Research: Reporting *In Vivo* Experiments” (ARRIVE) guidelines and approved by the animal experiment committees of Gifu Pharmaceutical University and Asahi University. All *in vivo* experimental procedures were performed using male ddY mice (four weeks old; body weight, 22 to 28 g; Japan SLC Ltd., Shizuoka, Japan), unless otherwise stated. Animals were housed at 24°C ± 2°C under a 12-h light–dark cycle. Food and water were available to all animals *ad libitum*.

### Focal cerebral ischemia-reperfusion in mice

Mice were anesthetized using 2.0 to 3.0% isoflurane, and maintained using 1.0 to 1.5% isoflurane in 70% N_2_O/30% O_2_, delivered via a facemask with an animal general anesthesia machine (Soft Lander, Sin-ei Industry Co., Ltd., Saitama, Japan). A midline skin incision was made in order to expose the left common carotid artery. The proximal portion of the common and external carotid arteries were ligated; thereafter, an 8–0 nylon monofilament (Ethicon, Somerville, NJ, USA), coated with a mixture of silicone resin (Provil novo, Heraeus Kulzer GmbH, Hanau, Germany), was introduced into the left internal carotid artery through the arteriotomy of the common carotid artery in order to obstruct the origin of the middle cerebral artery (MCA) [19,20]. After occlusion for 2 h, the nylon monofilament was gently withdrawn to restore blood flow in the MCA region. In each mouse, regional cerebral blood flow (rCBF) was monitored by laser Doppler flowmetry (Omegaflow flo-N1; Omegawave, Inc., Tokyo, Japan). Mice which did not demonstrate a significant reduction (to less than 40% baseline rCBF values) during middle cerebral artery occlusion (MCAO) were excluded. Sham control mice underwent the same surgical procedure, without obstruction of MCA. Body temperature of all animals was maintained at 37.0 to 37.5°C with the aid of a heating pad and heating lamp throughout these procedures. After the surgery, mice were housed under the preoperative conditions until further experimentation.

### Recombinant PGRN treatment

First, for dose–response studies, mice were randomly divided into four groups (n = 6 to 8 for each group). Two hours after the MCAO procedures, a Hamilton syringe was used to give each mouse a single intracerebroventricular (i.c.v.) injection, as has been described previously [21], containing 0.1, 0.3 or 1.0 ng of recombinant PGRN (r-PGRN; Recombinant Mouse Progranulin; R&D Systems, Inc., Minneapolis, MN, USA) in 2 μl of phosphatase-buffered saline (PBS); vehicle-treated control mice were injected with the same volume of PBS.

To investigate the long-term efficacy of r-PGRN treatment, mice were randomly divided into 1.0 ng r-PGRN-treated and vehicle-treated groups (n = 9 or n = 10 for each group). Their survival rates following MCAO were evaluated over a seven-day follow-up period.

For therapeutic time-window studies, mice were administered 1.0 ng of r-PGRN diluted in 2 μl PBS via i.c.v. administration 6 h after MCAO, while vehicle-treated control mice were injected with the same volume of PBS (n = 8 or n = 9 for each group).

For immunofluorescence staining and Western blot analysis of I/R tissue, mice were administered 1.0 ng of r-PGRN diluted in 2 μl PBS via i.c.v. administration 2 h after MCAO, while vehicle-treated control mice were injected with the same volume of PBS at the same time point (n = 4 or n = 5 for each group).

### Measurement of infarct and cerebral edema volumes

Mice were euthanized 24 h after the induction of focal cerebral ischemia. Their forebrains were quickly removed and sectioned coronally into five serial 2-mm slices. Tissue slices were placed in a 2% solution of 2, 3, 5-triphenyltetrazolium chloride (TTC; Sigma-Aldrich, St. Louis, MO, USA) at 37°C for 20 minutes, and then fixed in 10% buffered formalin. Digital images of the caudal aspect of each slice were obtained using a digital camera (Coolpix 4500, Nikon, Tokyo, Japan). The infarct, ipsilateral hemisphere and contralateral hemisphere areas were all measured using image-processing software (Image-J version 1.43 h; National Institutes of Health, Bethesda, MD, USA), and infarct volume was calculated as has been previously reported [19]. Cerebral edema was also calculated using the following formula: (infarct volume + ipsilateral undamaged volume – contralateral volume) × 100/contralateral volume (%) [20].

### Neurological deficit scoring

Mice were tested for neurological deficits 24 h after MCAO, and scored as described previously [19]. The possible scores were as follows: 0, no observable neurological deficits (normal); 1, failure to extend the right forepaw (mild); 2, circling to the contralateral side (moderate); and 3, loss of walking or righting reflex (severe). The investigator who rated the mice was blinded to their initial treatment condition.

### Immunofluorescence staining and cell counting in brain sections

Mice were anesthetized with an intraperitoneal injection of sodium pentobarbital (50 mg/kg, i.p.) 24 h after the induction of focal cerebral ischemia (n = 4 for each group), and perfused transcardially with 4% paraformaldehyde (PFA). The forebrain was removed, fixed in 4% PFA for 24 h and frozen. Fresh frozen forebrains were sliced into 12-μm-thick coronal sections by using a cryostat vibratome (Leica CM 1850; Leica Microsystems, Buffalo Grove, IL, USA), and sectioned tissues were placed onto individual slides. To identify infiltrating neutrophils on I/R brain slices, we performed immunohistochemistry for myeloperoxidase (MPO). Slides were blocked with 1% normal horse serum (Sigma-Aldrich) in PBS for 1 h at room temperature, and then incubated with the primary antibody for MPO (1:100; Abcam, Eugene, OR, USA) overnight at 4°C. The next day, slides were washed three times with PBS for 10 minutes each time, and then incubated with the secondary antibody Alexa Fluor 546 donkey anti-rabbit IgG (1:500; Molecular Probes, Eugene, OR, USA), for 1 h at room temperature. After three washes with PBS, the slides were incubated in Hoechst 33342 (1:10,000; Molecular Probes) for 10 minutes to provide nuclear counterstaining. Finally, slides were mounted using Vectashield fluorescent mounting medium (Vector Laboratory, Burlingame, CA, USA) and cover-slipped for microscopy. For quantitative analysis of cell number in the infarcted cortex and the corresponding region in sham animals, the slides were visualized and digitally photographed using a confocal microscope at a ×40 magnification (Fluoview FV-10; Olympus, Tokyo, Japan). Three fields of view were randomly chosen and photographed to count the number of MPO-positive cells in each section, and Image-J was used to analyze each picture. All analysis was performed blinded to the treatment condition.

### Western blot analysis

Expression levels of PGRN or MMP-9, and phosphorylation of nuclear factor-κB (NF-κB) in I/R brain were evaluated by Western blot analysis. I/R or sham control brain tissues (n = 4 or n = 5 for each group) were collected, and the brains were cut into 2-mm-thick coronal sections 6 to 8 mm from the frontal pole, and carefully separated into ipsilateral and contralateral hemispheres, with respect to the infarct location. The collected ipsilateral brain tissues were homogenized in lysis buffer (50 mM Tris–HCl (pH 8.0), containing 100 mM NaCl, 50 mM EDTA, 1% Triton X-100, and protease inhibitor cocktail (Sigma-Aldrich)) to extract the protein. Samples were centrifuged at 12,000 × *g* for 30 minutes at 4°C, and the supernatant collected. Equivalent amounts of total protein were separated by their molecular weights on SDS-PAGE gradient gel (SuperSep Ace; Wako Pure Chemicals, Osaka, Japan), and transferred to polyvinylidene difluoride (PVDF) membranes (Immobilon-P; Millipore Corporation, Billerica, MA, USA). Transfer was followed by blocking with 5% skimmed milk in TBS with 0.05% Tween-20 solution (TBS-T) for 30 minutes. Membranes were incubated overnight with primary antibodies at 4°C as follows: rat anti-PGRN (1:1,000, R&D Systems, Inc.), rabbit anti-MMP-9 (1:1,000, Millipore Corporation), rabbit anti-phosphorylated NF-κB (1:1,000, Cell Signaling Technology, Danvers, MA, USA), rabbit anti-total NF-κB (1:1,000, Cell Signaling Technology), and mouse anti-β-actin (1:5,000; Sigma-Aldrich). After three washes with TBS-T, membranes were incubated with the appropriate horseradish peroxidase-conjugated secondary antibodies (Thermo Fisher Scientific, Waltham, MA, USA) for 1 h at room temperature. After the final wash with TBS-T, immunoreactive bands were detected using a Lumino Imaging Analyzer (LAS-4000; Toyobo Engineering, Osaka, Japan). Signal intensity was measured using Image-J software, and normalized to the β-actin signal intensity.

### Gelatin zymography

MMP-9 activation was analyzed by gelatin zymography using a gelatin zymography kit (Cosmo Bio, Tokyo, Japan). I/R or sham control brain tissues (n = 3 for sham or n = 4 for each treated group) were collected, and tissue samples were lysated, homogenized and protein concentration was measured with Western blotting. Twenty micrograms of total proteins were subjected to electrophoresis in polyacrylamide gels containing 0.5 mg/mL gelatin in the presence of SDS under non-reducing conditions, washed twice in 2.5% Triton X-100 for 1 hour, rinsed briefly, and incubated at 37°C for 48 hours in 100 mmol/L Tris–HCl (pH 7.4) and 10 mmol/L CaCl2. Then they were stained with Coomassie Brilliant Blue R-250 and destained in a solution of 7.5% acetic acid and 5% methanol. Zones of enzymatic activity appeared as clear bands against a blue background; their signal intensity was measured using Image-J software.

### Neutrophil isolation and assay for binding of ^125^I-labeled TNF-α to the neutrophil surface

Male Wistar rats weighing 250 to 300 g (Japan SLC, Ltd.) were used for neutrophil isolation experiments. The animals were intraperitoneally injected with 25 to 30 mL of a 6.0% colloidal suspension of casein. Eighteen hours later, the animals were euthanized, and peritoneal exudates were collected from the peritoneal cavities. The samples were then centrifuged at 800 × *g* for 10 minutes at 4°C, and the resulting pellet was suspended in 5 mL of ice-cold hypotonic buffer (0.15 M NH_4_Cl, 1 mM KHCO_3_, 1 mM EDTA) and placed on ice for 10 s in order to remove contaminating erythrocytes. The neutrophils were then pelleted by centrifugation (800 × *g* for 10 minutes) and washed twice with 5 mL of PBS. This fraction, which contained over 95% viable neutrophils, was used for the following experiment [22]. ^125^Iodine-labeled recombinant human TNF-α was purchased from PerkinElmer, Inc. (Boston, MA, USA; specific activity 2.8 Bq/pg). Isolated neutrophils were seeded in six-well plates at a density of 1 × 10^5^ cells/well, and cultured for 1 h at room temperature with a constant amount of ^125^I-TNF-α (50 pg/mL) and different amounts of r-PGRN (between 0 and 250 ng/mL; R&D Systems, Inc.). For determination of nonspecific binding, cells were also incubated with ^125^I-TNF-α in the presence of a 500-fold excess of unlabeled TNF-α (Recombinant Human TNF-α; R&D Systems, Inc.). After incubation, cells were washed three times with cold PBS. The cells were subjected to a gamma scintillation counter. Specific binding was calculated by subtracting the nonspecific binding from the total binding. Data were obtained from three independent experiments.

### Neutrophil chemotaxis assay

Screening for neutrophil chemotaxis was performed using an EZ-TAXIScan chemotaxis apparatus (Effector Cell Institute, Kawasaki, Japan), as has been previously described [22]. Briefly, neutrophils were stimulated by the addition TNF-α (100 ng/ mL; Recombinant Mouse TNF-α; R&D Systems, Inc.) and different amounts of r-PGRN (between 0 and 250 ng/mL; R&D Systems, Inc.) to one hole, while treated cells were placed in a contra-hole of the microchannel. Migration of neutrophils toward the high concentration of each sample was recorded using a charge-coupled device (CCD) camera, with time-lapse images captured every 60 s for 60 minutes at 25°C with the EZ-TAXIScan apparatus. Sequential files and superimposed images of chemotaxing cells were processed using Motic Image Plus software (Shimadzu Corp., Kyoto, Japan). Viable cells were assessed in terms of their migration speed, straightness and directionality of cell movement, as previously described [23].

### Human microvascular endothelial cell culture and treatment

Human brain microvascular endothelial cells (hBMVEC) were purchased from Cell Systems (Kirkland, WA, USA). They were seeded at a density of 1 × 10^5^ cells per well onto 12-well culture plates, and incubated in EBM-2 medium with endothelial growth supplements (Lonza, Walkersville, MD, USA) at 37°C in 5% CO^2^ until they reached 70% confluence. To investigate the effects of PGRN on the TNF-α induced inflammation model, the culture medium was removed, and cells were washed twice with PBS, before they were treated with varying concentrations of recombinant human PGRN (rh-PGRN; Recombinant Human Progranulin; R&D Systems, Inc.), and 10 ng/mL of TNF-α (Recombinant Human TNF-α; R&D Systems, Inc.) for 20 h, in accordance with the methods described previously [24]. Following these treatments, cell lysates were collected, and Western blot analysis was performed in order to assess the expression levels of intercellular adhesion molecule-1 (ICAM-1), using the protocol described above, and the primary antibodies of rabbit anti-ICAM-1 (1:1,000; Cell Signaling Technology) and mouse anti-β-actin (1:5,000; Sigma-Aldrich).

### Statistical analysis

All values are expressed as mean ± SEM. The quantitative variables were statistically analyzed using Student’s two-tailed *t*-test for two-group comparisons, and a one-way ANOVA followed by Dunnett’s test for multiple pair-wise comparisons. A Wilcoxon signed-rank test was used for the repeated assessment of neurological scores, and a Log-rank test was used for the assessment of survival rates during follow-up periods. *P-*values of less than 0.05 were considered statistically significant. All statistical analyses were performed using JMP 7 for Macintosh (SAS Institute Inc., Cary, NC, USA).

## Results

### Expression of PGRN in the ischemia-reperfusion brain

**Figure 1:**
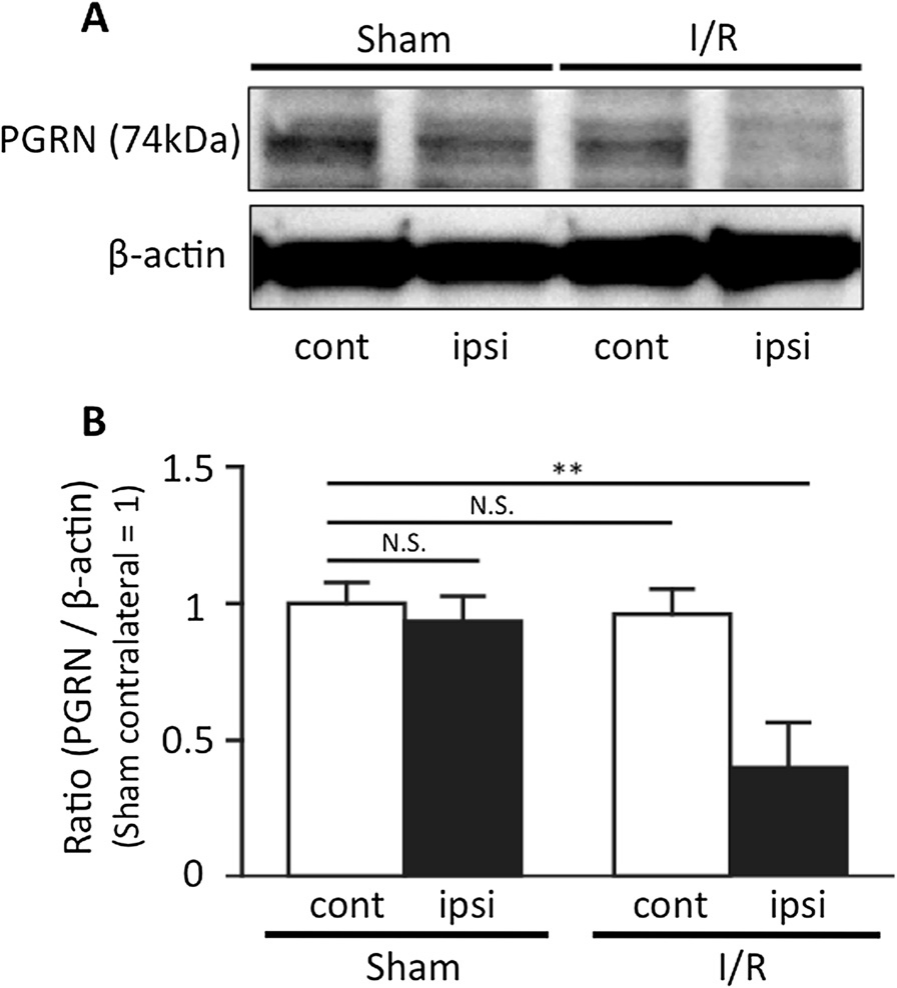
**Expression of progranulin in the ischemia-reperfusion injured brain.** Progranulin (PGRN) expression was significantly decreased following ischemia-reperfusion (I/R) insults. **(A)** Representative PGRN bands from the Western blotting analysis of brain tissue taken from sham-operated and I/R animals; ipsilateral and contralateral hemispheres to the middle cerebral artery occlusion (MCAO). **(B)** Optical densitometry quantification of PGRN protein levels, normalized to β-actin. In the I/R brain, the expression of PGRN was significantly decreased 24 h after the induction of transient cerebral ischemia. **P <0.01 vs. sham contralateral brain; one-way ANOVA followed by Dunnett's test; n = 4 for each group.

First, we examined the expression levels of PGRN in I/R brain at 24 h after the induction of focal cerebral ischemia. Interestingly, we found that PGRN expression was significantly decreased in I/R brain tissue. In the I/R brain, a 60% decrease in PGRN expression was observed compared to the sham contralateral, non-ischemic brain, 24 h after MCAO (Figure 1A,B; *P* <0.01 vs. sham contralateral brain; one-way ANOVA followed by Dunnett’s test).

### Efficacy of r-PGRN treatment on focal cerebral ischemia-reperfusion injury

**Figure 2:**
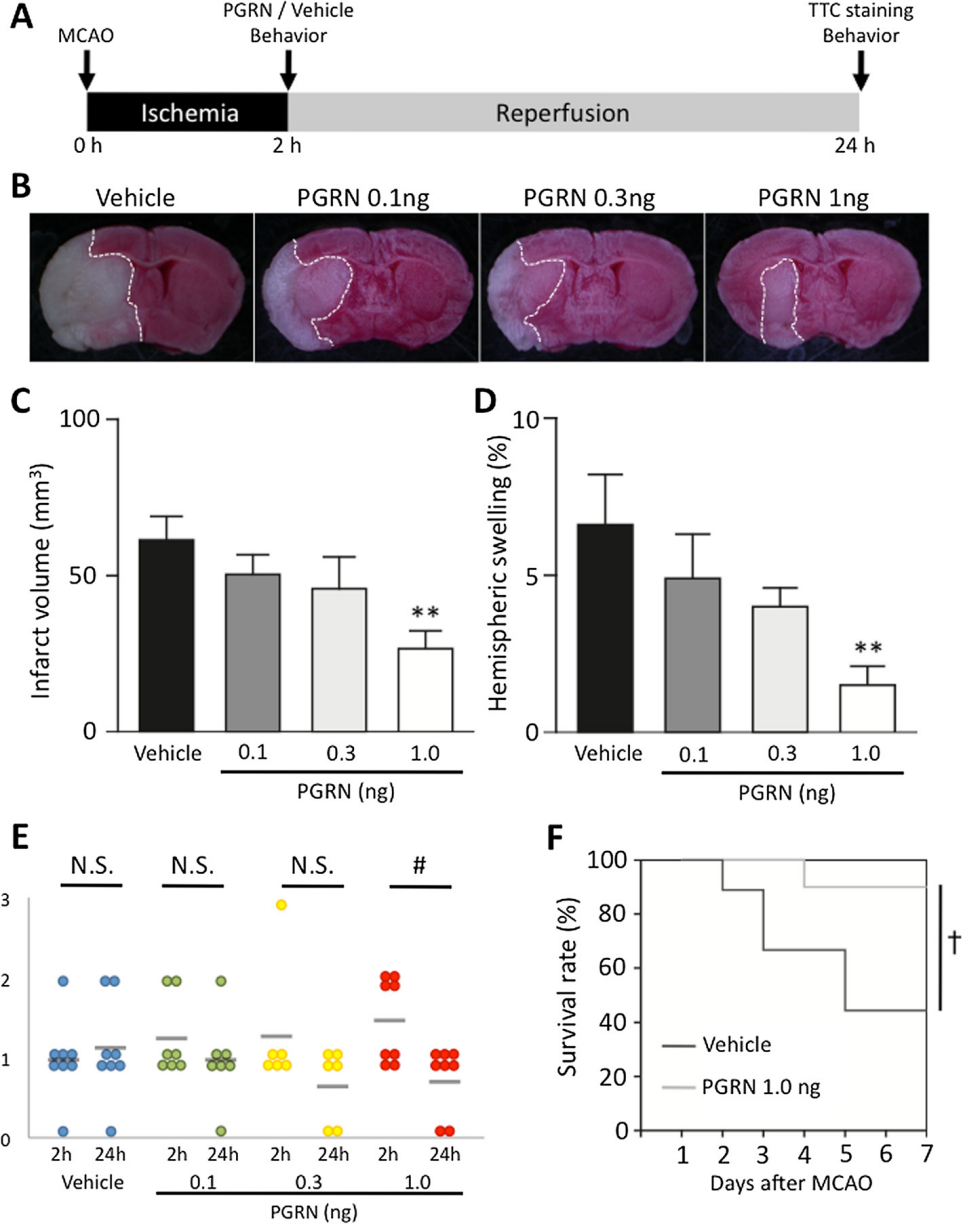
**r-PGRN treatment reduces cerebral infarct volume and brain edema in transient focal cerebral ischemia. (A)** Protocol for surgery and r-PGRN administration. Intracerebroventricular (i.c.v.) injections of either vehicle or r-PGRN (0.1 to 1.0 ng) were administered 2 h after middle cerebral artery occlusion (MCAO). All assessments, with the exception of survival rate evaluation, were performed at 24 h after the induction of 2 h of transient MCAO. **(B)** Representative photograph showing TTC staining of coronal brain sections 24 h after MCAO in each treatment group. **(C)** Administration of 1 ng of r-PGRN significantly reduced the infarct volume, **(D)** and reduced brain edema, compared to the vehicle treatment. Although the 0.1 ng r-PGRN- and 0.3 ng r-PGRN-treated groups tended to experience reduced infarct volume and brain edema, the difference was not statistically significant. * *P* <0.05 vs. vehicle-treated group; one-way ANOVA followed by Dunnett's test; n = 6 to n = 8 for each group. **(E)** Only the 1.0 ng r-PGRN-treated group had significantly better neurological function at 24 h after MCAO than at 2 h after MCAO. # *P* <0.05; Wilcoxon signed-rank test. **(F)** A higher survival rate was observed throughout the follow-up period in the 1.0 ng r-PGRN-treated group. In contrast, a continuous reduction of the survival rate was observed in the vehicle-treated group. The difference between the groups was statistically significant. † *P* <0.05; Log-rank test; n = 9 or n =10 for each group. r-PGRN, recombinant-progranulin.

Next, we examined whether the administration of r-PGRN would reduce infarct volume. The treatment protocol for this experiment is shown in Figure 2A. Mice developed an infraction affecting the left hemisphere after 2 h of MCAO, followed by 22 h of reperfusion; no mice, in any experimental group, died during this experiment. The administration of 1.0 ng of r-PGRN led to a 57% reduction in infarct volume (Figure 2B, 2C; *P* <0.01; one-way ANOVA followed by Dunnett’s test), and a 77% reduction in brain swelling, compared to the vehicle-treated group (Figure 2D; *P* <0.01; one-way ANOVA followed by Dunnett’s test). In the groups treated with 0.1 and 0.3 ng r-PGRN, we observed an 18% and 25% reduction in infarct volume, and a 26% and 40% reduction in brain swelling, respectively. However, there was no statistically significant difference between these groups and the vehicle-treated group. The neurological scores of each treated group 24 h after MCAO are shown in Figure 2E. Only the group treated with 1.0 ng r-PGRN had significantly better neurological function at 24 h after MCAO than at 2 h after MCAO (*P* <0.05; Wilcoxon signed-rank test). Figure 2F shows the survival rates of both the 1.0 ng r-PGRN-treated and vehicle-treated groups. The 1.0 ng r-PGRN-treated group had a high survival rate throughout the follow-up period (100% on Days 1 to 3; 90% on Days 4 to 7); in contrast, the vehicle-treated group showed a continuous reduction of survival rate from Day 2 (88.9%) until Day 7 (44.4%). There was a statistically significant difference between the two groups (*P* <0.05; Log-rank test).

### Therapeutic time-window for r-PGRN treatment

**Figure 3:**
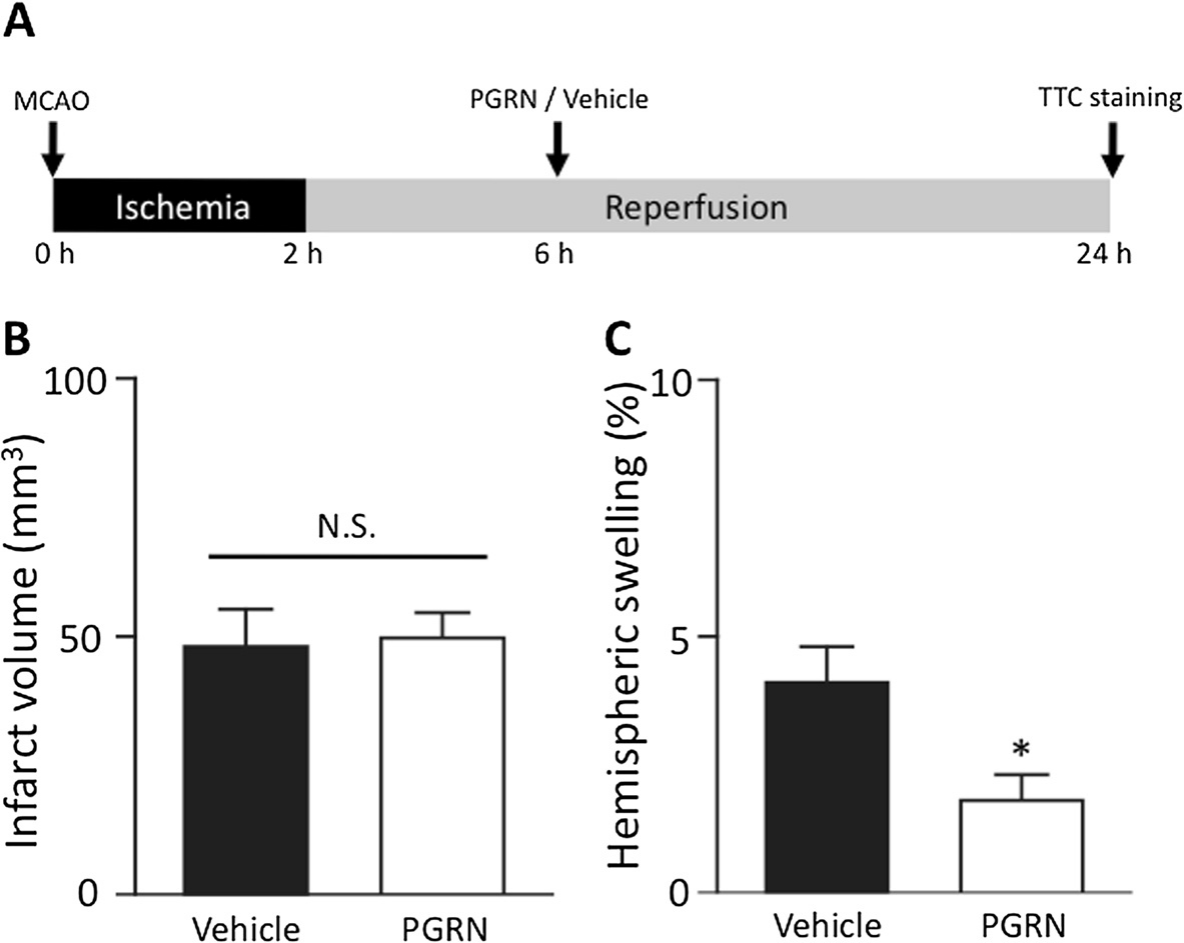
**The effects of delayed administration of r-PGRN 6 h after transient MCAO. (A)** Protocol for surgery and PGRN administration. Injections (i.c.v.) of either vehicle or r-PGRN (1.0 ng) were administered 6 h after the MCAO procedure. All assessments were performed at 24 h after the induction of 2 h of transient MCAO. **(B)** Administration of 1 ng of r-PGRN 6 h after MCAO did not reduce the infarct volume assessed at 24 h after the induction of 2 h of MCAO; **(C)** however, it significantly reduced brain edema. N.S. not significant; * *P* <0.05 vs. vehicle-treated group; Student's *t*-test; n = 8 or n = 9 for each group. i.c.v., intracerebroventricular; MCAO, middle cerebral artery occlusion; PGRN, progranulin; r-PGRN, recombinant-progranulin.

We also investigated the therapeutic time-window for r-PGRN treatment (the experimental protocol is shown in Figure 3A). Delayed administration of 1.0 ng of r-PGRN 6 h after MCAO did not reduce the infarct volume (Figure 2B); it did, however, lead to a 56% reduction of brain swelling compared to those of the vehicle-treated group (Figure 2C; *P* <0.05; Student’s *t*-test).

### r-PGRN attenuates neutrophil infiltration into I/R brain

**Figure 4:**
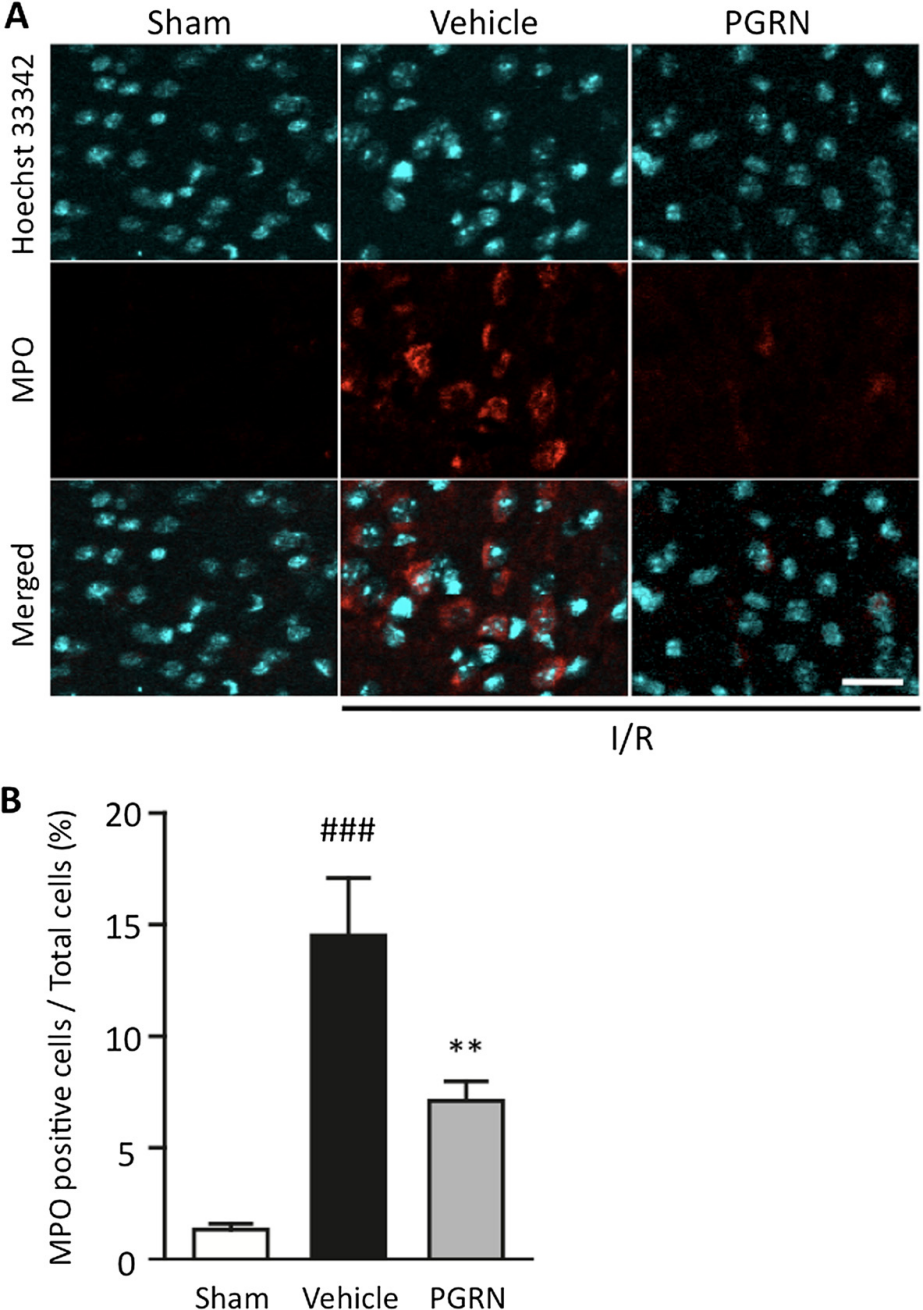
**r-PGRN treatment significantly suppresses neutrophil recruitment into the I/R brain following MCAO. (A)** Representative immunohistochemical staining for myeloperoxidase (MPO) in each of the areas of interest in the sham-operation, vehicle-treated and r-PGRN-treated groups. **(B)** Quantification of MPO-immunoreactive cells. The number of MPO-positive cells was significantly higher in the vehicle-treated mice than in the r-PGRN-treated mice. Scale bar = 20 μm. ## *P* <0.01 vs. sham-operation mice; ** *P* <0.01 vs. vehicle-treated mice; Student's *t*-test. n = 4 or n = 5 for each group. I/R, ischemia-reperfusion; MCAO, middle cerebral artery occlusion; r-PGRN, recombinant-progranulin.

It has been reported that neutrophils are the first leukocyte subpopulation to be recruited to the ischemic brain, and an extensive infiltration of neutrophils was observed 24 h after transient filament MCAO in mice [25]. We examined whether r-PGRN treatment inhibits neutrophil infiltration into the I/R brain. To identify infiltrating neutrophils, we stained the tissue for MPO. At 24 h after the induction of transient MCAO, the number of MPO-positive cells was found to be significantly increased in the vehicle-treated group (*P* <0.001 vs. sham operation control; Student’s *t*-test). Notably, the number of MPO-positive cells was significantly lower in the r-PGRN-treatment group than in the vehicle-treated group (*P* <0.01; Student’s *t*-test) (Figure 4A,B).

### PGRN acts as an antagonist to TNF-α and suppresses neutrophil chemotaxis

**Figure 5:**
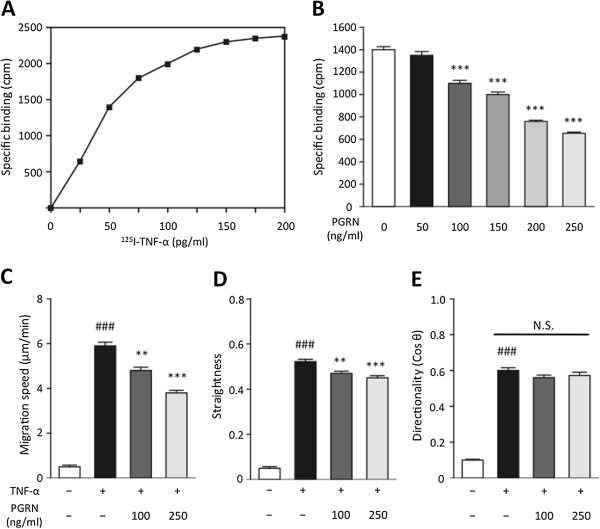
**PGRN inhibits **^**125**^**I-TNF-α binding to neutrophil surfaces and suppresses neutrophil chemotaxis induced by TNF-α. (A)** Saturation curve for specific ^125^I-TNF-α binding to neutrophil surfaces was determined, and in accordance with these results, 50 pg/mL of ^125^I-TNF-α was used in the subsequent experiments. **(B)** The ^125^I-TNF-α binding significantly decreased with increasing concentrations of PGRN. ***P <0.001 vs. 0 ng/mL of PGRN group; one-way ANOVA followed by Dunnett's test. Data were obtained from three independent experiments and presented as mean ± SEM. **(C, D, E)** Neutrophil chemotaxis was induced by TNF-α, and PGRN was found to significantly suppress this effect in a concentration-dependent manner; at 100 and 250 ng/mL of PGRN attenuates the migration speed and straightness of the route of migration, but did not affect the directionality of migration. ### *P* <0.001 vs. control group; Student *t*-test; ** *P* <0.01, *** *P* <0.001 vs. TNF-α only group; one-way ANOVA followed by Dunnett's test; n = 5 for each group. PGRN, progranulin; TNF-α, tumor necrosis factor-alpha.

First, the saturation curve for specific ^125^I-TNF-α binding to neutrophil surfaces was determined (Figure 5A); in accordance with these results, 50 pg/mL of ^125^I-TNF-α was used in the following experiments. ^125^I-TNF-α binding significantly decreased with increasing concentrations of PGRN, from 100 to 250 ng/mL (Figure 5B; *P* <0.001; one-way ANOVA followed by Dunnett’s test). These results strongly indicate that PGRN inhibits TNF-α/TNF-receptor interactions. Next, we investigated whether TNF-α causes neutrophil chemotaxis, and, if it does, whether PGRN suppresses the TNF-α-induced neutrophil chemotaxis. In these experiments, we found that neutrophil chemotaxis was indeed induced by TNF-α, and that PGRN significantly suppressed this chemotaxis in a concentration-dependent manner; doses of 100 and 250 ng/mL of PGRN significantly suppressed both neutrophil migration speed (Figure 5C; *P* <0.01, and *P* <0.001 vs. TNF-α only group, respectively; one-way ANOVA followed by Dunnett’s test) and the straightness of migration courses (Figure 5D; *P* <0.001 vs. TNF-α only group, for each dose; one-way ANOVA followed by Dunnett’s test). However, the directionality of migration was not significantly affected (Figure 5E).

### PGRN treatment reduces the expression of ICAM-1 in TNF-α-treated hBMVECs

**Figure 6:**
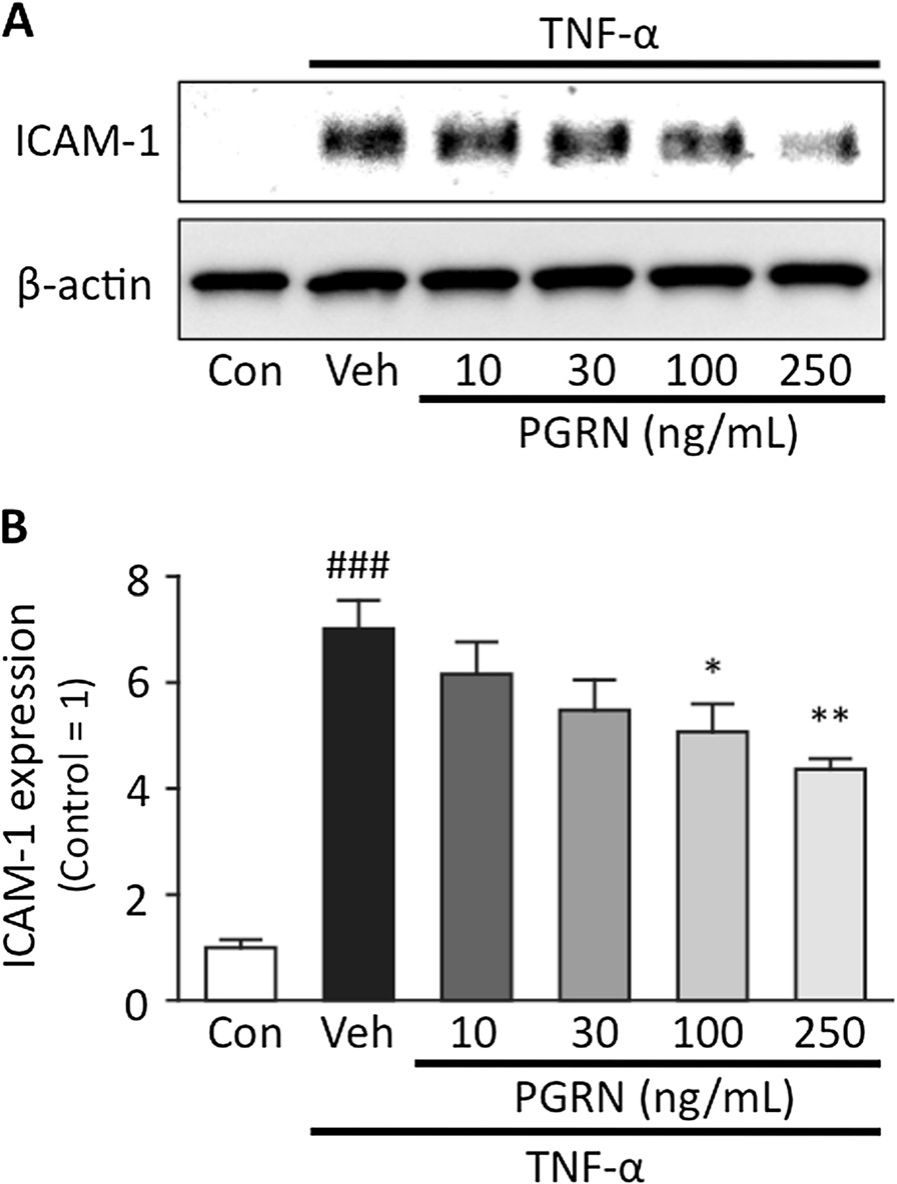
**PGRN ameliorates TNF-α-induced inflammation in hBMVECs. (A)** Representative bands from the Western blotting analysis of ICAM-1 and β-actin. **(B)** Optical densitometry quantification of ICAM-1, normalized to β-actin. TNF-α (10 ng/mL) induced an approximately eight-fold increase in ICAM-1 in hBMVECs after a 20-h exposure. ### *P* <0.001 vs. control group; Student's *t*-test. PGRN significantly suppressed TNF-α-induced ICAM-1 expression in a concentration-dependent manner. * *P* <0.05, ** *P* <0.01 vs. vehicle-treated group; one-way ANOVA followed by Dunnett's test; n = 4 for each group. hBMVECs, human brain microvascular endothelial cells; ICAM-1, intercellular adhesion molecule-1; PGRN, progranulin; TNF-α, tumor necrosis factor-alpha.

Proinflammatory cytokines induced by I/R facilitate the infiltration of leukocytes into brain tissue by activating and inducting adhesion molecules on vascular endothelial cells. In particular, intracellular adhesion molecule-1 (ICAM-1) plays an important role in the firm adherence of leukocytes [26]. In the present study, hBMVECs treated with TNF-α were used as an *in vitro* inflammatory model of brain endothelial cells. After 20 h of exposure to 10 ng/mL of TNF-α, ICAM-1 expression in the hBMVECs was significantly increased (*P* <0.001 vs. control group; Student’s *t*-test). This increased ICAM-1 expression was significantly attenuated by both 100 and 250 ng/mL of rh-PGRN, in a concentration-dependent manner (*P* <0.05 and *P* <0.01 vs. vehicle-treated group, respectively; one-way ANOVA followed by Dunnett’s test) (Figure 6A,B).

### Effects of r-PGRN on the phosphorylation of NF-κB, and expression, activation of MMP-9 in the I/R brain

**Figure 7:**
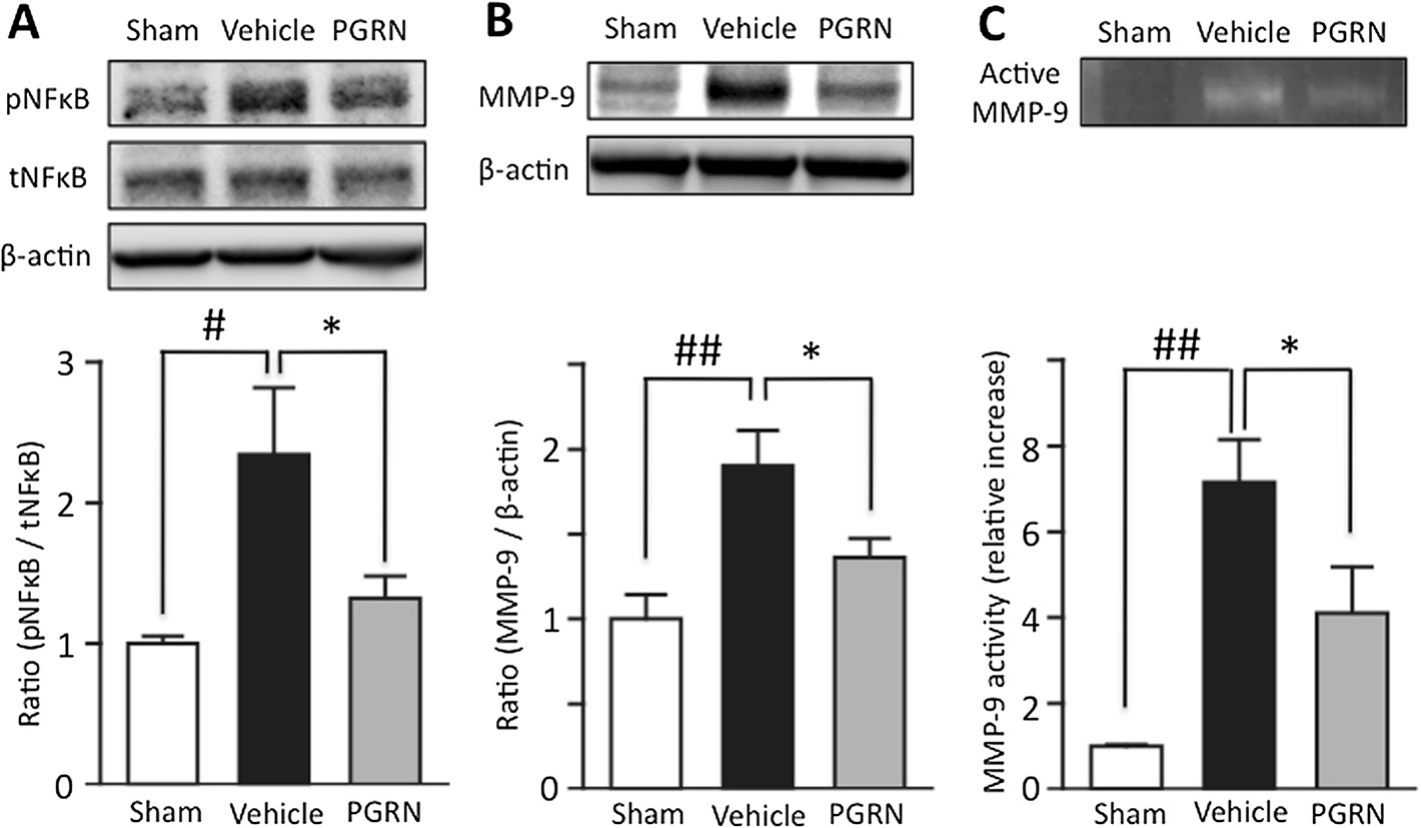
**PGRN significantly suppresses the expression of MMP-9, and the phosphorylation of NF-κB in I/R brain. (A)** Representative bands from Western blotting analysis of phosphorylated and total NF-κB (upper). Optical densitometry quantification for the phosphorylation of NF-κB (p NF-κB), normalized to total NF-κB (tNF-κB) and β-actin (lower). In the I/R brain, phosphorylation of NF-κB was significantly increased. ## *P* <0.01 vs. sham control group; Student's *t*-test. PGRN significantly suppressed this increased phosphorylation of NF-κB induced by I/R. * *P* <0.05 vs. vehicle-treated group; Student *t*-test. **(B)** Representative bands from Western blotting analysis of MMP-9 expression (upper). Optical densitometry quantification of MMP-9 expression, normalized to β-actin (lower). MMP-9 expression was significantly increased in the I/R brain. ## *P* <0.01 vs. sham control group; Student's *t*-test. PGRN significantly suppressed the expression of MMP-9 induced by I/R. * *P* <0.05 vs. vehicle-treated group; Student's *t*-test; n = 5 for each group. **(C)** Representative bands from gelatin zymography for activated MMP-9 (upper). Optical densitometry quantification of activated MMP-9 (lower). Activated MMP-9 was significantly increased in the I/R brain. ## *P* <0.01 vs. sham control group; Student's *t*-test. PGRN significantly suppressed the activation of MMP-9 induced by I/R. * *P* <0.05 vs. vehicle-treated group; Student's *t*-test; n = 3 for sham or n = 4 for each treated group. I/R, ischemia-reperfusion; MMP-9, matrix metalloproteinase-9; NF-κB, nuclear factor-κappaB; PGRN, progranulin.

The effects of r-PGRN treatment on the phosphorylation of NF-κB, and on the expression and the activation of MMP-9 24 h after the induction of transient focal ischemia are shown in Figure 6. In the I/R brain, the level of phosphorylated NF-κB was significantly increased in the vehicle-treated group (*P* <0.01 vs. the sham control group; Student’s *t*-test), while the level of total NF-κB did not differ between the groups. This increased level of NF-κB phosphorylation was significantly suppressed by r-PGRN treatment (*P* <0.05 vs. vehicle-treated group; Student’s *t*-test) (Figure 7A). The expression of MMP-9 was significantly increased in the vehicle-treated group (*P* <0.05 vs. sham control group; Student’s *t*-test), and this increase was suppressed by r-PGRN treatment (*P* <0.05 vs. vehicle-treated group; Student’s *t*-test) (Figure 7B). Additionally, activated MMP-9 detected by gelatin zymography was significantly increased in the vehicle-treated group (*P* <0.001 vs. sham control group; Student’s *t*-test), and this increase was suppressed by r-PGRN treatment (*P* <0.05 vs. vehicle-treated group; Student’s *t*-test) (Figure 7C).

## Discussion

Many previous studies have indicated a role for PGRN in the pathogenesis of chronic CNS disorders. In the CNS, PGRN is expressed in both neurons and microglia [27], and it is well recognized that a decreased level of functional PGRN, due to null or missense mutations of the *PGRN* gene, causes frontotemporal lobar degeneration, with ubiquitin-positive inclusions [12,17,28]. PGRN is secreted as a glycosylated polypeptide, and is thought to exert at least some of its biological functions within the extracellular space; it is considered to play an important role in neuronal tissue homeostasis [29]. However, the biological functions of PGRN in case of acute neuronal injury remain unclear.

In this study, we found that the expression of PGRN was significantly decreased in the I/R brain 24 h after the induction of transient focal cerebral ischemia (Figure 1). It is known that full-length PGRN acts as an anti-inflammatory agent; however, its derivative, granulin, acts in the opposite manner, stimulating the production of proinflammatory cytokines [30]. During an inflammatory reaction, neutrophils and macrophages secrete the protease elastase that digests full-length PGRN into individual 6-kDa granulin peptides, potentially exacerbating the inflammation [30]. It has been suggested that PGRN inhibits the neutrophil activation and recruitment triggered by proinflammatory mediators in the initial stages of inflammation. However, as the level of proinflammatory mediators rise, neutrophil elastase (NE) levels, secreted by activated neutrophils, increase and NE degrades PGRN into granulin. Subsequently, neutrophils release further proinflammatory mediators and chemotactic agents, enhancing the recruitment of further neutrophils and exacerbating inflammation [31]. We, therefore, hypothesized that decreased levels of PGRN potentiates the neuroinflammation induced by I/R, and that its mechanisms are, at least in part, due to promotion of neutrophil recruitment and activation.

In the present study, we demonstrated that the administration of r-PGRN significantly attenuated neuronal injury following I/R, with a 6-h therapeutic time-window (Figures 2 and 3). Recently, Tao *et al.* reported that transgenic mice over-expressing PGRN had smaller cerebral infarctions and better functional outcomes after focal cerebral ischemia than wild-type mice [18]. They also showed that the expression of proinflammatory cytokines was significantly lower in astrocytes cultured from PGRN-over-expressing mice. However, they did not fully elucidate the anti-inflammatory mechanisms of PGRN. Experimentally and clinically, focal cerebral ischemia induces the recruitment and activation of inflammatory cells, including various types of leukocytes [6,7]. Among the various leukocytes, neutrophils are the first to infiltrate into the ischemic brain, and neutrophil infiltration is recognized as an important pathogenic factor following a cerebral ischemic insult [32]. Neutrophil infiltration into the brain tissue was found to be more prominent in transient, but not in permanent, ischemia in the early phase [25,33], and occurred within 30 minutes to a few hours, peaking within the first three days [4,9]. In our study, consistent with the findings of previous studies, a marked increase in neutrophil infiltration following I/R insult was observed in the infarct cortex in the vehicle-treated group. r-PGRN treatment significantly suppressed this neutrophil infiltration (Figure 4), with these results suggesting that r-PGRN treatment attenuates the neuronal damage caused by I/R through the suppression of harmful neutrophil recruitment.

In the earliest phase of cerebral ischemia, TNF-α is released predominantly from microglia [4,5,34], and plays a critical role in subsequent I/R-induced injury. It has been suggested that TNF-α primes neutrophil extravasation from blood vessels during inflammation [31]. More recently, it was reported that PGRN binds directly to TNF receptors and suppresses TNF-α-mediated inflammation in a mouse model of rheumatoid arthritis [15]. To the best of our knowledge, we are the first to report that PGRN directly inhibits TNF-α binding to neutrophils, and to confirm that PGRN significantly suppresses the neutrophil chemotaxis triggered by TNF-α in a concentration-dependent manner, as demonstrated by an *in vitro* assay (Figure 5). These results suggest that PGRN is a potentially useful candidate for the attenuation of TNF-α-mediated inflammation.

TNF-α is considered to be a major mediator of inflammatory responses in vascular endothelial cells [24]. Cell-adhesion molecules, particularly ICAM-1, are induced during the early stages of ischemia by TNF-α, along with other proinflammatory cytokines [35,36]; subsequently, leukocytes begin to firmly adhere to endothelial cells, from where they can infiltrate into the brain tissue (Smith *et al.* 1998; Stanimirovic *et al.* 1997). To determine the effects of PGRN on endothelial inflammation, we used hBMVECs, which we exposed to TNF-α, as an *in vitro* model of endothelial inflammation, in accordance with previous literature [24]. In this model, co-treatment with PGRN significantly reduced TNF-α-induced ICAM-1 expression in a concentration-dependent manner (Figure 6). These results indicate that PGRN has dual mechanisms of suppressing neutrophil recruitment, one through the direct inhibition of neutrophil chemotaxis, and the other, by ameliorating endothelial inflammation. Additionally, in the I/R brain, TNF-α may directly affect neuronal or glial cells by binding TNF receptors and up-regulating inflammatory signals. Previous studies have suggested that neurons express both TNF-receptor1 (TNF-R1) and 2 (TNF-R2) [37], and that TNF-R2 signaling plays a larger role in inflammatory responses following stroke [5]. It was reported that PGRN had higher binding affinity for TNF-R1 and TNF-R2, especially TNF-R2, when compared to TNF-α [15]. Taken together, these findings suggest that PGRN potentially attenuates the neuronal inflammation caused by TNF-α. Although anti-inflammatory approaches targeting neutrophils or ICAM-1 have proved to be successful in animal models, attempts to transfer this knowledge to a clinical setting have thus far been unsuccessful [7]. In comparison with these approaches, PGRN treatment seems to be more promising with regard to clinical applications because of its multiple anti-inflammatory effects on neutrophils, vascular endothelium and neuronal cells.

Finally, we confirmed that r-PGRN treatment significantly reduces the phosphorylation of NF-κB and the expression of MMP-9 in the I/R brain (Figure 7). Expression and activation of MMP-9 following cerebral ischemia are closely associated with disruption of the blood–brain barrier (BBB), and cause severe brain edema or hemorrhagic transformation [38]. Although post-ischemic MMP-9 expression was increased in neurons, glia, endothelial cells and infiltrated neutrophils, recruited neutrophils are considered the key cellular source of MMP-9, which promotes further recruitment of neutrophils in a positive feedback manner and causes BBB disruption [39]. Hence, inhibition of neutrophil recruitment by PGRN also suppresses MMP-9 expression by terminating the abovementioned positive feedback mechanism, and thus ameliorates BBB disruption.

NF-κB is an oxidative stress-responsive transcription factor, and its involvement in I/R injury is well recognized [40]. In the early phase of post-cerebral I/R, infiltrating neutrophils cause excessive production of ROS, resulting in oxidative stress in the affected brain tissue. Oxidative stress promotes the activation of NF-κB and its translocation to the nucleus, where activated NF-κB mediates the transcription of various inflammatory genes, thus inducing inflammation. In this study, we found a significant reduction in phosphorylated NF-κB levels in r-PGRN-treated mice. Because excessive amounts of ROS are produced by recruited neutrophils in the I/R brain [11], it is reasonable to speculate that the inhibition of neutrophil recruitment by PGRN leads to the suppression of NF-κB activation and ameliorates inflammation via the NF-κB pathway.

## Conclusion

The potentially beneficial effects of PGRN in ischemic stroke have been confirmed, using both *in vivo* and *in vitro* experimental models of cerebral I/R injury. These effects are, at least in part, due to anti-inflammatory mechanisms and, specifically, the inhibition of neutrophil infiltration. The current findings indicate the feasibility of r-PGRN treatment as a novel anti-inflammatory therapy, which may prove beneficial in the acute stage of ischemic stroke.

## Abbreviations

ARRIVE: Animal research: Reporting *In Vivo* experiments; BBB: Blood–brain barrier; CNS: Central nervous system; FBS: Fetal bovine serum; hBMVECs: Human brain microvascular endothelial cells; ICAM-1: Intercellular adhesion molecule-1; i.c.v.: Intracerebroventricular; i.p.: Intraperitoneal; I/R: Ischemia-reperfusion; MCA: Middle cerebral artery; MCAO: Middle cerebral artery occlusion; MMP: Matrix metalloproteinase; MPO: Myeloperoxidase; NE: Neutrophil elastase; NF-κB: Nuclear factor-κappaB; PBS: Phosphate-buffered saline; PFA: Paraformaldehyde; PGRN: Progranulin; rCBF: Regional cerebral blood flow; PVDF: Polyvinylidene difluoride; ROS: Reactive oxygen species; r-PGRN: Recombinant-progranulin; TBS-T: Tris-buffered saline with 0.05% Tween-20 solution; TNF-α: Tumor necrosis factor-α; TNF-R1: TNF-receptor1; TNF-R2: TNF-receptor2.

## Competing interests

The authors declare that they have no competing financial or personal interests, and that none of the authors’ institutions has contracts relating to this research through which it may stand to gain financially now or in the future.

## Authors’ contribution

YE and HH were responsible for study conception and design. YE drafted the article. KT, MS, YS, MK, IT and HH critically revised the manuscript for important intellectual content. YE, YS, YA, TT, SS and KM acquired the data. YE, SY, YA, TT, SS, KM, KT and MS analyzed and interpreted the data. SY, MK, IT and HH supervised the study. All authors read and approved the final manuscript.
